# Columbia suicide severity rating scale screen scores in adults with Chiari malformation Type 1

**DOI:** 10.1371/journal.pone.0334599

**Published:** 2025-10-16

**Authors:** Richard Labuda, Emme Nolan, Emily P. Rabinowitz, Douglas L. Delahanty, Petra M. Klinge, Philip A. Allen

**Affiliations:** 1 Conquer Chiari, Wexford, Pennsylvania, United States of America; 2 Department of Psychology, University of Akron, Akron, Ohio, United States of America; 3 Department of Psychological Sciences, Kent State University, Kent, Ohio, United States of America; 4 Department of Neurosurgery, Warren Alpert Medical School of Brown University, Rhode Island Hospital, Providence, Rhode Island, United States of America; University of Kansas School of Medicine Wichita, UNITED STATES OF AMERICA

## Abstract

Adult Chiari malformation Type 1 is a neurological condition characterized by high levels of chronic pain, disability, and psychological distress, yet self-harming behaviors have not previously been studied in this patient group. The purpose of this study was to determine the prevalence of elevated suicide risk scores among adults with Chiari malformation Type I using the Columbia Suicide Severity Rating Scale – Screen and examine the association between risk scores and pain, disability, and psychological distress. A web-based, anonymous survey was administered comprised of validated scales and demographic questions. Suicide risk (Columbia Screen score) was dichotomized as Low or High-Risk and both crude and adjusted odds ratios were calculated to determine statistical associations with pain, disability, and psychological measures. Overall, 44% of 372 respondents scored in the High-Risk group. Logistic regression showed that depression at the moderate level or above (Adjusted Odd Ratio (AOR) = 4.27, 95% CI = 2.58–7.05, p < .0001), age younger than 30 years (AOR = 3.10, 95% CI = 1.67–5.78, p = .0003), and severe or complete neck related disability (AOR = 2.02, 95% CI = 1.22–3.33, p = .0056) were significant predictors of High-Risk scoring. This study is the first to examine suicidal ideation and risk in the adult CMI population. Clinicians should be aware that suicidal ideation is a serious morbidity in this patient population.

## Introduction

Symptomatic, adult Chiari Malformation Type I (CMI) is a neurological condition characterized by high levels of chronic pain, disability, and depression, yet self-harming and suicidal behaviors have not yet been studied in this patient group [1–3]. Clinically, CMI involves herniation of the cerebellar tonsils through the foramen magnum at the bottom of the skull resulting in direct compression of neural tissue and disruption of the natural flow of cerebrospinal fluid across the cranio-vertebral junction [4,5]. Common symptoms include headaches (acute Valsava induced and chronic migraine type), neck pain, shoulder pain, balance issues, depression, anxiety, cognitive impairment, and trouble sleeping [6–9]. For reasons that have not yet been elucidated, close to 80% of adult CMI patients are female [10]. A subset of patients (~54%) [6] undergo neurosurgery to create more room around the compressed tissue and restore the natural flow of cerebrospinal fluid [5]. However, this procedure has limited long-term outcomes, and patients are often left with residual physical, psychological, and functional issues [1,2,11]. Although it has not been previously studied, both surgical and non-surgical CMI patients could be at elevated risk of self-harming and suicidal behaviors due to high levels of chronic pain, disability, and depression [1–3,12–14].

Specifically, it has been shown that 80% of adult CMI women have moderate-complete neck related disability as measured by the Neck Disability Index (NDI) [2]. In addition, pain levels reported by CMI patients on the Short Form McGill Pain Questionnaire 2 (SF-MPQ-2) are comparable to what is found in other chronic pain conditions [3]. Interestingly, both the disability and pain findings are independent of surgical status [2,3]. Meanwhile, Meeker et al. [15] found that more than 90% of adult CMI patients struggle with one or more aspects of daily living. Psychologically, Garcia et al. [1] found that 44% of adult CMI patients suffer from moderate-severe depression and 60% from moderate-severe anxiety (Depression, Anxiety and Stress Scale – 21, DASS-21), again independent of surgical status. Finally, a large survey found that 78% of patients still employed found it difficult to work due to CMI, and of those not employed 79% said it was due to CMI [6].

Both chronic pain and depression have been well demonstrated as major risk factors for self-harmful behaviors up to and including suicide [12,13]. In addition, a nationally representative survey of nearly 200,000 subjects found that people with functional disabilities were significantly more likely to report suicidal ideation, planning, and attempts than the general population [14].

The Columbia Suicide Severity Rating Scale – Screening Version (C-SSRS Screen) is a shortened version of the widely used Columbia-Suicide Severity Rating Scale [16]. Bjureberg et al. [16] evaluated the utility of the C-SSRS Screen in predicting 7-day, one month, and one year completed suicide in more than 18,000 consecutive psychiatric emergency room patients and found the C-SSRS Screen score was significantly associated with higher odds of completed suicide at all three time points.

The purpose of this cross-sectional study was to quantify the suicide risk among a relatively large sample (N = 372) of CMI adults (65% post-surgical) using the C-SSRS Screen administered through a web-based anonymous survey, and further to examine the relationship between the level of risk with pain, disability, and psychological distress.

We hypothesized that CMI adults would exhibit high levels of suicide risk and that pain, disability, and psychological distress would be significant, independent predictors of the C-SSRS Screen scores.

## Materials and methods

The research was approved by the Institutional Review Board at The University of Akron (#2022065), and all participants provided written, electronic informed consent. A web-based, anonymous survey was constructed using *Qualtrics* (Provo, UT: Qualtrics; 2022). The survey consisted of demographic and Chiari history questions, plus eight validated scales to assess suicide risk, pain, disability, and mental health.

Given the nature of the study, the participant debrief included instructions on how to contact the National Suicide Prevention Lifeline as well as a web link for additional resources. While the study of suicidal ideation and behaviors raises valid ethical concerns, Gould et al. found in a large, randomized control trial of high school students that screening did not increase distress levels, ideation, or attempts [17]. In a review of the literature, Dazzi et al. also found no evidence that screening increased ideation [18].

### Subjects

Adults diagnosed with CMI were recruited to participate in a web-based study on the “Psychological Impact of Chiari” using a large patient organization’s (Conquer Chiari) website, email list, and social media platforms. Respondents under 18 years at the time of the survey, those without a physician diagnosis of CMI (self-reported), and those who did not complete all the scales were excluded from this analysis (the C-SSRS Screen was presented last in the survey). Between 9/6/2022–12/12/2022, 520 people responded at least in part to the survey. Out of these, 372 (71.5%) met the inclusion criteria and completed all survey elements.

### C-SSRS screen

The C-SSRS Screen, which was designed to be administered without training by anyone,evaluates suicide risk based on five progressive, previous month ideation focused questions and one behavior history question [16]. The responses are then categorized as No, Low, Medium, or High-Risk, with good internal reliability (Cronbach’s α = 0.89) [19]. Bjureberg et al. [16] found using an area under the curve analysis that the optimal cut-off to predict suicide in the subsequent week, month, and year was ideation with methods, which equates to the Medium Risk category.

### Additional survey measures

The UCLA Loneliness Scale is a 20-item assessment which uses a 4-point scale (1 = never to 4 = always, with certain items reverse scored) to measure feelings of loneliness and social isolation (Cronbach’s α = 0.89–0.94) [20]. The DASS-21 is a widely used instrument comprised of depression, anxiety, and stress subscales with 7 questions each scored on a Likert scale (Cronbach’s alpha = 0.91) [21,22]. The NDI is the most widely used, validated instrument for assessing neck-related disability and consists of ten questions covering daily activities like personal care and work [23]. Each item is rated from 0 (no disability) to 5 (complete disability), with total scores doubled to represent a percentage disability (Cronbach’s α = 0.86) [24]. The SF-MPQ-2 assesses pain across four domains: continuous, intermittent, neuropathic, and affective [25]. Respondents rate 22 items on an 11-point Likert scale (0 = none to 10 = worst). The scale shows high reliability for subscales (Cronbach’s α = 0.73–0.89) and total scores (α = 0.91–0.94). The Impact of Events Scale-Revised (IES-R) measures trauma-related psychological distress using 22 items and a 5-point Likert scale (0 = not at all to 4 = extremely, Cronbach’s α = 0.79–0.95) [26]. The Pain Catastrophizing Scale (PCS) assesses negative thinking about pain through 13 items on a 5-point Likert scale (0 = not at all to 4 = all the time, Cronbach’s α = 0.92) [27].

Given the time period during which the study took place, a COVID Impact Scale was included to account for potential confounding effects of the pandemic. The Coronavirus Impact Scale (CIS) measures how much the pandemic changed the respondent’s life in terms of daily routine, access to food, mental health, etc. The instrument uses a 4-point Likert scale (0 = No change to 4 = Severe, Cronbach’s α = 0.90–0.96) [28].

### Demographics and surgical history

The survey also included questions on gender, age, and Chiari surgical history. Race and ethnicity data were not collected to ensure anonymity. In our previous experience with similar surveys, respondents were overwhelmingly white and non-Hispanic, therefore we did not want to risk exposing the identity of any minority respondents.

### Statistical analysis

The dependent variable was the C-SSRS Screen score dichotomized as Low-Risk (None-Low) or High-Risk (Medium-High) (note the terms Low and High-Risk as used throughout this paper mean based on the C-SSRS Screen score, not a clinical diagnosis). Independent variables included Age, Gender, Chiari Surgical History, Loneliness (UCLA), Pain (SF-MPQ-2 Total), Depression (DASS-21 subscale), Anxiety (DASS-21 subscale), Neck Related Disability (NDI), Impact of Events (IES Revised), Pain Catastrophizing (PCS), and COVID Impact (CIS).

Means and standard deviations were computed for the continuous variables, and frequency and percentages for the categorical variables. Student’s t-tests and chi-square tests were used to compare subjects who scored at High-Risk vs Low-Risk across all independent variables.

The continuous independent variables were then categorized using either published cut-off points (Loneliness, Depression, Anxiety, Neck Related Disability, Post Traumatic Stress Disorder (PTSD based on IES), Pain Catastrophizing) [21,23,27,29,30] or quartiles (Pain, COVID Impact) if no categorical interpretation was available in the literature. Age was categorized by decade up to 50 years. The categorical variables were then used to calculate crude Cochran-Mantel-Haenszel odds ratio estimates and 95% confidence intervals of scoring at High-Risk.

Two logistic regression models were constructed with High-Risk as the outcome variable. The first model (Continuous) utilized the scale values in their continuous form to identify significant predictors while adjusting for confounding. To assist with interpretation, a second logistic model (Binary) was constructed based on the results of the first model (which variables to include) and the crude odds ratios (the lowest categorical level with a significant OR relative to the reference level was set as a dichotomization point). The second model used Age dichotomized at 30 years, Depression dichotomized at moderate, and Neck Disability dichotomized at severe as the input variables To account for potential collinearity, a linear regression model was used with the C-SSRS risk score as the output variable and the input variables in continuous form where applicable was constructed to determine the variance inflation factor (VIF) of each input variable. P-values below.05 (two-tailed) were considered significant.

All statistical analyses were performed using *SAS* (Version 9.4. Cary, NC: SAS Institute; 2022) and *Microsoft Excel for Microsoft 365* (Version 2201. Redmond, WA: Microsoft Corp.; 2022).

## Results

The average age of the study sample was 43 years and 92.7% were female. Sixty-five percent had undergone Chiari surgery. Overall, 162 (43.5%) participants were in the High-Risk group based on the CSSRS-Screen (Table 1). The High-Risk group was 5 years younger on average than the Low-Risk group (p = .0005) but the differences in gender and Chiari surgical history were not significant. The High-Risk group scored significantly higher on every scale measure, with all but Loneliness (p = .0046) maintaining significance even after correcting for multiple observations (p < .0045).

**Table 1:** Demographic, functional, and psychological distress measures of adult CMI patients scoring at high (N = 162) and low (N = 210) risk on the C-SSRS screen.

	High-Risk Mean (SD) or n (%)	Low-Risk Mean (SD) or n (%)	p value^c^
Age (years)^a^	40.5 (12.5)	45.0 (12.2)	.0005
Gender^b^			.6441
Male	8 (4.9)	15 (8.1)	
Female	152 (95.1)	193 (91.9)	
Chiari Surgical History			.2893
No	61 (37.7)	68 (32.4)	
Yes	101 (62.3)	142 (67.6)	
Loneliness (UCLA)	57.0 (4.6)	55.7 (4.5)	.0046
Depression (DASS-21)	24.0 (11.4)	14.8 (12.0)	<.0001
Anxiety (DASS-21)	18.6 (10.2)	13.6 (10.6)	<.0001
Neck Related Disability (NDI)	45.0 (18.4)	37,1 (17.5)	<.0001
Pain (SF-MPQ-2 Total)	116.8 (44.8)	94.6 (49.7)	<.0001
Impact of Events (IES)	40.9 (17.6)	31.9 (18.1)	<.0001
Pain Catastrophizing (PCS)	30.2 (12.1)	21.2 (13.7)	<.0001
COVID Impact (CIS)	9.7 (5.4)	7.9 (5.6)	.0017

^a^ One missing value in High-Risk group.

^b^ Three responded Other, one missing value in High-Risk group.

^c^ Student’s t-test or χ^2^.

Categorizing the variables (Table 2) demonstrated that the odds of scoring at High-Risk for suicide decreased significantly with older age, with participants aged 30 years and older having less than half the odds of being High-Risk as those aged 18–29. Participants with moderate Depression were more than three times as likely to be in the High-Risk group as those who scored in the normal range (OR = 3.50, 95% CI = 1.79–6.86), while participants who scored as extremely severe in Depression were more than six times as likely to be High-Risk (OR = 6.56, 95% CI = 3.60–11.94). Severe Neck Disability was significantly associated with High-Risk status (OR = 4.71, 95% CI = 1.62–13.73), as was Pain at every quartile above the first. Participants with IES-R scores suggestive of PTSD were twice as likely to be in the High-Risk group (OR = 2.38, 95% CI = 1.56–3.64), while those meeting the cut-off for Pain Catastrophizing were three times as likely to be in the High-Risk group (OR = 3.2, 95% CI = 2.08–4.93).

**Table 2:** Univariate odds ratio analysis for scoring at high-risk on C-SSRS screen among adults with CMI (N = 372).

Factor	C-SSRS Screen Risk Category				
	n (%)			OR (95% CI)	p value
	Total	High (2–3)	Low (0–1)		
Age (years)^a^					
18-29	62 (16.8)	39 (62.9)	24 (37.1)	1.00 (Ref)	
30-39	81 (21.8)	35 (43.2)	46 (56.8)	0.48 (0.24–0.94)	.0327
40-49	117 (31.5)	50 (42.7)	67 (57.3)	0.47 (0.25–0.88)	.0185
50+	111 (29.9)	38 (34.2)	73 (65.8)	0.33 (0.17–0.63)	.0006
Gender^b^					
Male	23 (6.3)	8 (34.8)	15 (65.2)	1.00 (Ref)	
Female	345 (93.7)	152 (44.1)	193 (55.9)	1.48 (0.61–3.57)	.386
Chiari Surgical History					
No	129 (34.7)	61 (47.3)	68 (52.7)	1.00 (Ref)	
Yes	243 (65.3)	101 (41.6)	142 (58.4)	0.79 (0.52–1.22)	.29
Loneliness (UCLA)^c^					
Low-Moderate (20–49)	27 (7.2)	7 (25.9)	20 (74.1)	1.00 (Ref)	
High (50–64)	331(89.0)	145 (43.8)	186 (56.2)	2.23 (0.92–5.41)	.0711
Very High (64–80)	14 (3.8)	10 (71,4)	4 (28.6)	7.14 (1.69–30.28)	.0056
Depression (DASS-21)					
Normal (0–9)	109 (29.3)	22 (20.2)	87 (79.8)	1.00 (Ref)	
Mild (10–12)	36 (9.7)	10 (27.8)	26 (72.2)	1.52 (0.64–3.62)	.3425
Moderate (13–20)	66 (17.7)	31 (47.0)	35 (53.0)	3.50 (1.79–6.86)	.0002
Severe (21–27)	44 (11.8)	26 (59.1)	18 (40.9)	5.71 (2.67–12.23)	<.0001
Extremely Severe (28–42)	117 (31.5)	73 (62.4)	44 (37.6)	6.56 (3.60–11.94)	<.0001
Anxiety (DASS-21)					
Normal (0–6)	93 (25.0)	22 (23.7)	71 (76.3)	1.00 (Ref)	
Mild (7–9)	28 (7.5)	13 (46.4)	15 (53.6)	2.80 (1.16–6.77)	.0203
Moderate (10–14)	80 (21.5)	35 (43.8)	45 (56.2)	2.51 (1.31–4.81)	.0052
Severe (15–19)	39 (10.5)	16 (41.0)	23 (59.0)	2.25 (1.01–4.98)	.0451
Extremely Severe (20–42)	132 (35.5)	76 (57.6)	56 (42.4)	4.38 (2.43–7.90)	<.0001
Neck Related Disability (NDI)					
None (4–8)	23 (6.2)	5 (21.7)	18 (78.3)	1.00 (Ref)	
Mild (10–28)	77 (20.7)	28 (36.4)	49 (63.6)	2.06 (0.69–6.14)	.1928
Moderate (30–48)	156 (41.9)	61 (39.1)	95 (60.9)	2.31 (0.82–6.55)	.1081
Severe (50–68)	97 (26.1)	55 (56.7)	42 (43.3)	4.71 (1.62–13.73)	.0027
Complete (70+)	19 (5.1)	13 (68.4)	6 (31.6)	7.80 (1.95–31.15)	.0026
Pain (SF-MPQ-2)					
Quartile 1 (0–69)	92 (24.7)	25 (27.2)	67 (72.8)	1.00 (Ref)	
Quartile 2 (70–102)	95 (25.5)	43 (45.3)	52 (54.7)	2.22 (1.20–4.09)	.0103
Quartile 3 (103–143)	91 (24.5)	44 (48.4)	47 (51.6)	2.51 (1.35–4.65)	.0032
Quartile 4 (144+)	94 (25.3)	50 (53.2)	44 (46.8)	3.05 (1.65–5.62)	.0003
PTSD (IES Revised)					
No (0–32)	173 (46.5)	56 (32.4)	117 (67.6)	1.00 (Ref)	
Suspected (33+)	199 (53.5)	106 (53.3)	93 (46.7)	2.38 (1.56–3.64)	<.0001
Pain Catastrophizing (PCS)					
No (<30)	221 (59.4)	71 (32.1)	150 (67.9)	1.00 (Ref)	
Yes (31+)	151 (40.6)	91 (60.3)	60 (39.7)	3.20 (2.08–4.93)	<.0001
COVID Impact (CIS)					
Quartile 1 (0–4)	95 (25.5)	31 (32.6)	64 (67.4)	1.00 (Ref)	
Quartile 2 (5–8)	100 (26.9)	41 (41.0)	59 (59.0)	1.43 (0.80–2.58)	.2273
Quartile 3 (9–12)	85 (22.8)	36 (42.4)	49 (57.6)	1.52 (0.83–2.78)	.1792
Quartile 4 (13–24)	92 (24.8)	54 (58.7)	38 (41.3)	2.93 (1.62–5.33)	.0004

^a^ Missing one value.

^b^ Missing one value; three responded ‘Other’.

^c^ Low and Moderate categories combined due to no responses in Low category.

The collinearity check showed all VIF < 3, indicating collinearity among the input variables would not influence the regression models. The first regression model (Continuous) demonstrated good concordance (c = .76), with Age, Depression, and Neck Disability as the only significant predictors (p < .05) when controlling for all other variables (Table 3). The second logistic regression model (Binary) also demonstrated good concordance (c = .73), with Depression at the moderate level or above having the highest adjusted odds ratio of being in the High-Risk group (AOR = 4.27, 95% CI = 2.58–7.05). In addition, those under the age of 30 years had three times the odds of being in the High-Risk group (AOR = 3.10, 95% CI = 1.67–5.78), and those with severe or complete Neck Related Disability had twice the odds of being in the High-Risk group (AOR = 2.02, 95% CI = 1.22–3.33).

**Table 3:** Logistic regression models with continuous and selected binary factors for C-SSRS screen high-risk score among adults with CMI (N = 371).

*Continuous Model (c = .76)*	AOR (95% CI)	p value
Age (years)	0.97 (0.95 - 0.99)	.0021
Gender (Male)	0.75 (0.28 - 2.01)	.6674
Chiari Surgical History (Yes)	0.77 (0.47 - 1.26)	.8383
Loneliness (UCLA)	1.05 (0.99 - 1.10)	.0819
Depression (DASS-21)	1.06 (1.03 - 1.09)	<.0001
Anxiety (DASS-21)	0.97 (0.94 - 1.00)	.0775
Neck Related Disability (NDI)	1.02 (1.00 - 1.04)	.0391
Pain (SF-MPQ-2)	1.00 (0.99 - 1.01)	.8459
Impact of Events (IES Revised)	1.00 (0.98 - 1.02)	.8266
Pain Catastrophizing (PCS)	1.02 (0.99 - 1.04)	.2163
COVID Impact (CIS)	1.03 (0.98 - 1.07)	.2223
***Binary Model (c = .73)***		
Young Age	3.10 (1.67 - 5.78)	.0003
Depressed	4.27 (2.58 - 7.05)	<.0001
Neck Disability	2.02 (1.22 - 3.33)	.0056

c = concordance statistic; AOR = adjusted odds ratio; CI = confidence interval.

## Discussion

This cross-sectional study found that 44% of adults with CMI were at an elevated risk of suicide as defined by the CSSRS-Screen. In addition, being under the age of 30, having moderate or higher depression (DASS-21), or severe to complete neck related disability (NDI) significantly increased the odds of being classified as High-Risk for suicide. However, gender and history of Chiari surgery were not related to suicide risk.

Suicide risk has not been previously studied in the CMI population. However adult CMI patients exhibit several factors linked to increased risk of suicidal behavior, including high levels of depression, chronic pain, and disability [1–3,12–14]. In a review of the epidemiology of suicide, Bachmann [12] noted, “Depression is the leading cause of death by suicide worldwide…half of all completed suicides are related to depressive and other mood disorders.” In a separate review, Lepine and Briley [31] found that the mortality risk for suicide among depressed patients is more than twenty times greater than the general population. Our study found that depression (at the moderate or higher level on the DASS-21) was a strong, independent predictor of being classified in the High-Risk group.

Chronic pain is also a major risk factor for suicidal behaviors [13,32]. In a review, Tang and Crane [13] found that relative to controls the risk of death is double for chronic pain patients and the lifetime prevalence of suicidal ideation is around 20%. Calati et al. [32] found in a meta-analysis that individuals with any type of pain were significantly more likely to experience death wish, suicidal ideation, planning, attempts, and completions. They concluded that physical pain is a consistent risk factor for suicidal thoughts and behaviors. While our study showed that on average participants in the High-Risk group reported pain scores that were 23.5% higher than the Low-Risk group (p < .0001), pain was not an independent, significant predictor in the regression models.

In a secondary analysis of data collected through the 2020 Nations Survey on Drug Use and Health, Marlow et al. [14] found that people with disabilities were more than twice as likely to report suicidal ideation, planning, and attempts. Our study found similar results, with participants who reported severe to complete neck related disability being twice as likely to be in the High-Risk group. While these categories represent high levels of disability, they are common among adult CMI patients. Specifically, 31% of the study sample were classified as such, and in a separate study of 474 adult females with CMI, Labuda et al. [2] found that 44% reported severe to complete neck related disability.

The present study also found that older participants had significantly lower odds of scoring High-Risk compared to younger participants. This is counter to global, general population suicide rates where older adults (age 65+) are the most at risk age group, especially men [33]. This could be due in part to the composition of the study group, with only 4% of participants being 65 years or older and with no men in this category. A post-hoc analysis demonstrated a weak but significant negative correlation between age and both depression (r = −.13, p = .0149) and anxiety (r = −.18, p = .0007). However, this seems unlikely to provide a full explanation of this result, especially since all three variables were included in the regression modeling. Further study may be warranted to elucidate the nature of the association.

CMI patients often seek care from a number of providers during the course of their diagnosis and clinical care, including neurosurgeons, neurologists, and pain specialists. However, based on our experience, screening for depression and/or suicide risk is not routinely performed for CMI patients. The results of our survey suggest that suicidal ideation rates are unusually high in both surgically treated and untreated Chiari patients, and that the CSSRS-Screen may be a useful tool to identify those at risk. Our data further supports the importance of a study of suicide risk and its clinical and health implications in the CM1 patient population.

The present study has two main limitations. First, the cross-sectional design precludes any conclusions regarding causality. While several predictor variables showed strong statistical associations with an elevated risk score on the C-SSRS Screen, the precise nature of the associations cannot be definitively determined and requires additional research.

Second, because participants were recruited through a patient organization’s website and social media, the study sample may include selection bias which could affect the accuracy of the point estimates and potential generalizability. Specifically, it is possible that the study sample may include subjects with more severe symptoms and/or worse outcomes than the general Chiari population. The surgical rate – which can be used as a crude surrogate for disease severity in the group – was 65% in the study sample compared to 54% in a much larger sample [6]. However, previous research and this study’s results indicate that suicide risk scores, neck related disability, and depression are all comparable among surgical and non-surgical patients [1,2], suggesting that the impact of a surgically skewed bias would be minimal. While clinic-based studies of suicide risk would be valuable, the logistics of performing a comparable study to the present one would be challenging. First, not many clinical centers have the patient flow for a study this size, but more importantly it is our experience that most surgical CMI patients are only followed clinically for 12 months after surgery. Therefore, a clinic-based sample would only be looking at a small window of time in the patient experience and would not be representative of the general CMI population.

It is worth noting that the potential effects of delivering the C-SSRS screen anonymously through the web are not known. However, it should be emphasized that the Screen was designed using plain language and intended for use by anyone without any training. It seems reasonable that the anonymous, remote format may actually result in more accurate responses than in-person assessments, since the respondents are not concerned with the potential consequences of their answers, and thus more willing to disclose any ideation or previous suicide attempts. Finally, it is important to note that the study data was collected leading up to the holiday season which could have biased the C-SSRS Screen scores. However, 92.2% of the responses were collected prior to 10/31, so the magnitude of any such bias is likely minimal.

## Conclusion

Forty-four percent of adults with CM1 surveyed were at an elevated risk of suicidal behavior as measured by the C-SSRS Screen, independent of surgical treatment. Age under 30 years, depression at the moderate level or worse as scored on the DASS-21, and severe to complete neck related disability significantly increased the odds of scoring at higher risk. This study is the first to examine suicidal ideation and risk in the adult CMI population.
